# Senotherapeutic Effect of Retinaldehyde and Hyaluronate Fragments in Dermatoporosis

**DOI:** 10.3390/dermatopathology10020024

**Published:** 2023-06-02

**Authors:** Aysin Kaya, Jean-Hilaire Saurat, Gürkan Kaya

**Affiliations:** 1 Department of Clinical Pharmacology and Toxicology, University of Geneva, 1211 Geneva, Switzerland; 2 Department of Dermatology, University Hospital of Geneva, 1211 Geneva, Switzerland

**Keywords:** senotherapy, senolytics, senomorphics, p16^Ink4a^, retinaldehyde, hyaluronate fragments, dermatoporosis

## Abstract

Cellular senescence is one of the important mechanisms of skin aging. In a recent study, we have shown that in patients with dermatoporosis, an extreme senescence condition of the skin, cells positive for p16^Ink4a^, a biomarker of senescence, were significantly increased in the epidermis. Senescent cells can develop a senescence-associated secretory phenotype (SASP) comprising pro-inflammatory cytokines, chemokines, and other soluble factors, leading to chronic inflammation and tissue dysfunction. These senescent cells and SASP pathways represent therapeutic targets for the development of senotherapeutics either by inducing selective cell death of senescent cells called senolytics, or suppressing markers of the SASP, called senomorphics. In this study where we conducted a retrospective immunohistochemical analysis of p16^Ink4a^ expression in the skin samples of dermatoporosis patients included in a previous clinical study, we describe the senotherapeutic effect of retinaldehyde (RAL) and intermediate-size hyaluronate fragments (HAFi). Topical application of RAL and HAFi significantly reduced the number of p16^Ink4a^-positive cells in the epidermis and dermis in dermatoporosis patients which also showed a significant clinical improvement.

## 1. Introduction

One of the hallmarks of aging is the accumulation of senescent cells in tissues. The number of senescent cells increases in chronological aging and age-related pathologies [1].

Cell-cycle inhibitor p16^Ink4a^ encoded by *Cdkn2a* has been regarded as an indicator of cellular senescence since it has been shown to be expressed in senescent cells in different tissues. Senescent cells are defined as cells permanently arrested in the cell cycle with morphological changes, upregulation of senescence-associated β-galactosidase (SA-β-Gal), and a senescence-associated secretory phenotype (SASP) characterized by the secretion of chemokines, cytokines, proteases, and growth factors. Senescent cells acquiring SASP become harmful to the neighboring cells rendering them dysfunctional [2].

The number of p16^Ink4a^-positive cells in human skin was shown to be a marker of biological age. In addition, p16^Ink4a^ cellular senescence in situ is associated with age-related pathologies. Clearance of p16^Ink4a^-positive cells in a mouse model delayed the onset of age-related diseases [3].

In 2007 we proposed the term “dermatoporosis” to describe a chronic cutaneous insufficiency syndrome, a new dimension of skin aging beyond cosmetics and appearance, to explore its molecular mechanisms, and to develop preventive or therapeutic modalities. Dermatoporosis has recently turned out to be a prevalent skin condition recognized by the European Academy of Dermatology and Venereology. Prominent morphological markers of dermatoporosis are skin atrophy, clinically characterized by wrinkled skin, noninflammatory senile purpura, and pseudoscars. These markers are usually seen at around 70 years of age in chronically sun-exposed body areas but can also appear at earlier ages as a result of chronic systemic or topical corticosteroid therapy [4].

**Figure 1:**
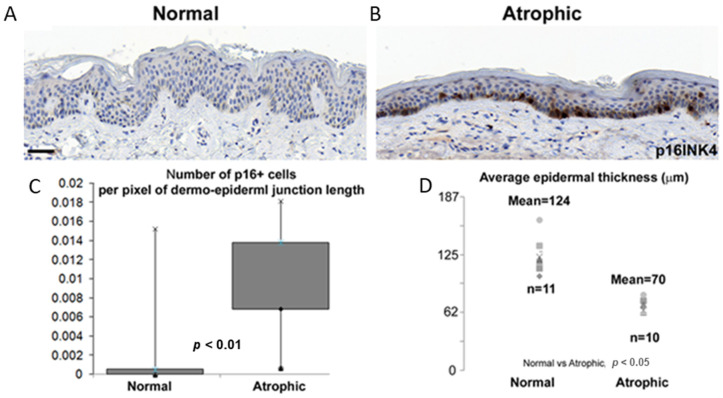
p16^Ink4a^ staining in the epidermis of healthy subjects (**A**) and dermatoporosis patients (**B**). The quantification of p16^Ink4a^ for each group was calculated by dividing the number of p16-positive cells by the dermal–epidermal junction length in the average of 3 microscope fields per subject (**C**), and the quantification of the epidermal thickness (the distance between the granular layer and the dermal–epidermal junction) was calculated by dividing the epidermal area by the length of the dermal–epidermal junction in the average of 3 microscope fields per subject (**D**). Note the epidermal atrophy and the significant increase of p16-positive cells in dermatoporosis patients (figure modified from [5]).

In a recent study, we showed that in 10 patients with dermatoporosis, p16^Ink4a^-positive cells were significantly increased in the epidermis (Figure 1) [5].

In that study, we had used the skin samples of 11 healthy subjects (mean age: 32 years; SD = 9; anatomic locations: 10 arms, and 1 forearm) and 10 dermatoporosis patients (mean age: 76 years; SD = 13; anatomic locations: 4 forearms, 2 arms, 1 neck, 3 cheek). These samples had been previously collected for diagnostic purposes and obtained from the histopathology laboratory of the Dermatopathology Unit of the University Hospital of Geneva according to the authorization CER12-091 and guidelines of the Ethical Commission on Human Research of the University Hospital of Geneva. Some of the skin samples had been the re-excision material of tumor-free specimens with no residual tumor and no pathological alterations in the nonlesional skin. All skin samples had been treated in a blinded fashion by the investigators; a trained dermatopathologist (GK) had selected the dermatoporotic skin and evaluated the p16^Ink4a^ immunostaining [5].

## 2. Results

**Figure 2:**
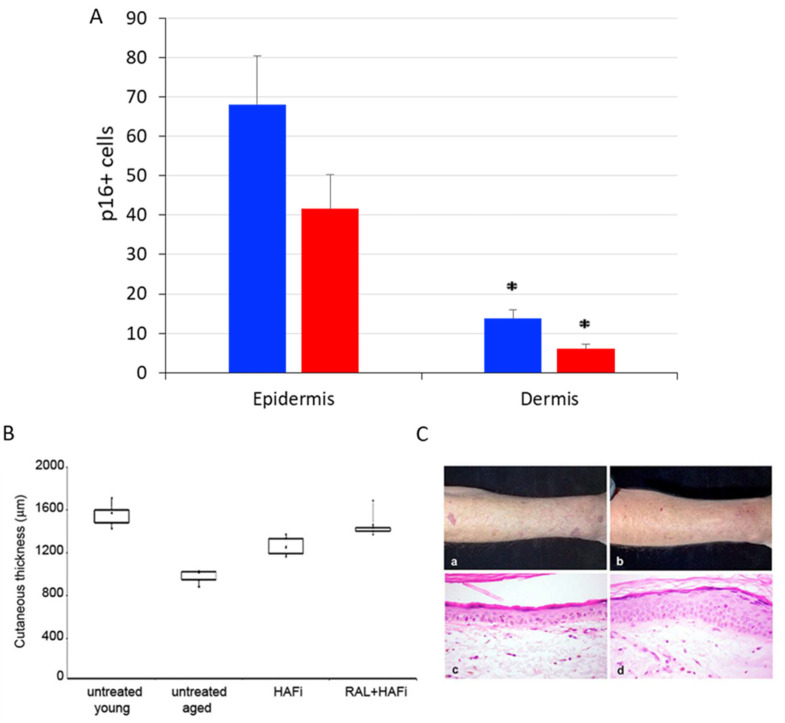
p16^Ink4a^ positive cells were significantly decreased after RAL and HAFi application in the epidermis and dermis (blue: before the treatment, red: after the treatment) (asterisk = *p* < 0.05, Student’s *t* test) (**A**). Skin ultrasonography of these patients showed a significant increase in skin thickness (measured between the upper limit of the epidermis and the dermal–subcutaneous fat junction) after RAL and HAFi treatment accompanied by a clinical improvement (**B**,**C**). This effect was more significant than RAL alone or HAFi alone. The results are presented as boxplots with median value (point in the boxes). *p* = 0.001 (untreated young versus untreated dermatoporosis); *p* < 0.001 (untreated dermatoporosis versus dermatoporosis treated with RAL and HAFi (nonparametric Mann–Whitney U test) (**B**). Clinical aspect of dermatoporotic forearm skin before (**a**) and 1 month after topical treatment with RAL and HAFi (**b**). Note the decrease of atrophy, purpuric lesions, and pseudoscars, and after RAL and HAFi treatment. The histological aspect of dermatoporotic forearm skin before (**c**) and 1 month after topical treatment with RAL and HAFi (**d**). Note the significant epidermal hyperplasia, decrease of elastosis, and increase of collagen content and vascularity in the dermis after RAL and HAFi treatment (**C**) (low panel modified from [6]).

In this current study, we have retrospectively analyzed the number of p16^Ink4a^-positive cells by immunohistochemistry in the forearm skin samples of dermatoporosis patients after topical treatment with retinaldehyde (RAL) and intermediate-size hyaluronate (HA) fragments (HAFi), which had been included in a previous clinical study. The p16^Ink4a^ positive cells were significantly reduced in the epidermis and dermis 1 month after RAL and HAFi application. These patients had also showed a significant clinical improvement with an increase in skin thickness measured by ultrasonography (Figure 2) [6].

## 3. Materials and Methods

Seven healthy young adults (mean age 25.5 years; SD = 7; anatomic location: forearm) and six patients with dermatoporosis (mean age: 78 years; SD = 10, anatomic location: forearm) had been included in a clinical study after obtaining written informed consent. This study had been conducted according to the authorization CER10-029 and guidelines of the Ethical Commission on Human Research of the University Hospital of Geneva. All the subjects had been topically treated with a combination of 0.05% RAL and 1% HAFi cream samples of 0.5 g on the forearm twice daily for 30 days. Forearm-skin biopsies of patients had been performed before and 1 month after application [6]. The skin samples which had been fixed in 10% formalin and embedded in paraffin blocks were re-used in this study. The samples were cut into 5-µm sections, and stained with anti-p16^Ink4a^ antibodies (rabbit monoclonal, 1:1000, ab108349, Abcam).

## 4. Discussion

Our previous studies have shown that the topical application of HAFi reversed skin atrophy in dermatoporosis patients by a CD44-dependent mechanism [7]. CD44 is the main cell-surface receptor for HA and is present on a membrane platform called hyalurosome along with other molecules involved in the metabolism of HA and cell signaling such as heparin-binding epidermal growth factor (HB-EGF), HB-EGF receptor ErbB1 and hyaluronate synthase-3 (HAS3), located in keratinocyte filopodia [6]. Specific suppression of CD44 in keratinocytes leads to skin atrophy in transgenic mice, suggesting that CD44 plays an important role in the regulation of epidermal homeostasis [8]. We have also demonstrated that RAL and HAFi show a synergistic effect on skin hyperplasia in mouse and human skin and seem to have a therapeutic effect in dermatoporosis [6]. Furthermore, our studies indicated that the topical RAL and HAFi combination regulates the expression of hyalurosome platform genes and shows a dose-dependent effect on the reversal of skin atrophy in dermatoporosis patients [9].

The senescence of skin cells is an important feature of skin aging; however, we have demonstrated the advanced stage of skin aging by the increased p16^Ink4a^ immunostaining of epidermal cells for the first time in dermatoporosis patients. Therefore, the clinical improvement observed by the application of the RAL and HAFi combination raised the possibility of elimination of the senescent cells residing in the epidermis.

The fact that the clearance of p16^Ink4a^-expressing cells in BubR1-hypomorphic progeroid mice delays aging-associated disorders and the results of other studies suggested that elimination or weakening of the function of senescent cells may be a promising approach for age-related pathologies. These strategies are collectively named ‘senotherapies’ [10]. There are two kinds of senotherapeutics: senolytics, which induce senolysis in senescent cells, and senomorphics, which attenuate their pathological proinflammatory secretory phenotype to cause senostasis [11]. Each senotherapeutic modality has various advantages and disadvantages. Many senolytic agents including synthetic small molecules and peptides have been developed for in vitro and in vivo [12].

Recent clinical trials conducted with senolytic agents in age-related human disease showed significant clinical improvement. In the first clinical trial of senolytic agents, Dasatinib and Quercetin decreased physical dysfunction in patients with idiopathic pulmonary fibrosis, a senescence-associated disease [13]. In another clinical trial, Dasatinib administration to patients with systemic sclerosis reduced the SASP and other senescence markers in skin biopsies [14]. Dasatinib and Quercetin, used in another clinical trial, significantly decreased senescent cells in patients with diabetic kidney disease [15]. There are currently other ongoing or planned senotherapeutic clinical trials [16].

## 5. Conclusions

The elimination of senescent cells by senolysis or by attenuation of SASP using senolytic or senomorphic molecules is an attractive novel strategy in skin aging and age-related skin diseases. Our results showing the decrease of p16^Ink4a^ expression in the skin samples of dermatoporosis patients after RAL and HAFi treatment in this retrospective study indicate that RAL and HAFi can be used as effective senotherapeutic agents in dermatoporosis. Further studies are required to understand the molecular mechanisms of senolysis or senostasis in the reversal of skin aging and consequently to develop new therapeutic strategies.

## Data Availability

Not applicable.
